# Design of a novel integrated microfluidic chip for continuous separation of circulating tumor cells from peripheral blood cells

**DOI:** 10.1038/s41598-022-20886-1

**Published:** 2022-10-11

**Authors:** Maliha Saleem Bakhshi, Mohsin Rizwan, Ghulam Jilany Khan, Hong Duan, Kefeng Zhai

**Affiliations:** 1 Mechatronics and Control Engineering Department, University of Engineering and Technology, Lahore, Pakistan; 2 Department of Pharmacology and Therapeutics, Faculty of Pharmaceutical Sciences, University of Central Punjab, Lahore, Pakistan; 3 School of Biological and Food Engineering, Engineering Research Center for Development and High Value Utilization of Genuine Medicinal Materials in North Anhui Province, Suzhou University, Suzhou, Anhui 234000 China; 4 Key Laboratory for Chemistry and Molecular Engineering of Medicinal Resources (Guangxi Normal University), Guilin, 541004 People’s Republic of China

**Keywords:** Biological techniques, Biotechnology, Cancer, Medical research, Engineering, Nanoscience and technology

## Abstract

Cancer is one of the foremost causes of death globally. Late-stage presentation, inaccessible diagnosis, and treatment are common challenges in developed countries. Detection, enumeration of Circulating Tumor Cells (CTC) as early as possible can reportedly lead to more effective treatment. The isolation of CTC at an early stage is challenging due to the low probability of its presence in peripheral blood. In this study, we propose a novel two-stage, label-free, rapid, and continuous CTC separation device based on hydrodynamic inertial focusing and dielectrophoretic separation. The dominance and differential of wall-induced inertial lift force and Dean drag force inside a curved microfluidic channel results in size-based separation of Red Blood Cells (RBC) and platelets (size between 2–4 µm) from CTC and leukocytes (9–12.2 µm). A numerical model was used to investigate the mechanism of hydrodynamic inertial focusing in a curvilinear microchannel. Simulations were done with the RBCs, platelets, CTCs, and leukocytes (four major subtypes) to select the optimized value of the parameters in the proposed design. In first stage, the focusing behavior of microscale cells was studied to sort leukocytes and CTCs from RBCs, and platelets while viable CTCs were separated from leukocytes based on their inherent electrical properties using dielectrophoresis in the second stage. The proposed design of the device was evaluated for CTC separation efficiency using numerical simulations. This study considered the influence of critical factors like aspect ratio, dielectrophoretic force, channel size, flow rate, separation efficiency, and shape on cell separation. Results show that the proposed device yields viable CTC with 99.5% isolation efficiency with a throughput of 12.2 ml/h.

## Introduction

According to the World Health Organization (WHO) and Global Cancer Observatory, the number of new cancer cases per year is expected to rise to 29.5 million and the number of cancer-related deaths to 16.4 million by 2040^1,2^. Most of the times cancer is not diagnosed and treated until tumor cells have metastasized throughout the body^3^. Subsequently patient relapses shortly with a very little survival rate. Circulating Tumor Cells (CTCs) are the cells that shed from primary tumors, recurrences, or metastases, and circulate in the peripheral blood, and possess antigenic and genetic tumor-specific characteristics^4^. CTCs may give rise to secondary growth of tumors at other sites of the body called metastases^4^. CTCs have distinct morphological and molecular characteristics; they start showing up in whole blood at a very early stage of tumor development^5^. It has been found that disease diagnosis, monitoring, and personalized cancer therapy can be done by keeping an account of CTCs^6^. Considering the fact, it is essential to detect and assess rare CTCs (a non-invasive marker) to diagnose the disease early enough for effective treatment^7^. Enumeration of CTCs from whole blood is very challenging due to their scarcity in patients at early stages of cancer i.e., 1–10 cells/ml^8^ as compared to other cells 5 × 10^9^/ml RBCs, 2 × 10^8^/ml platelets, and 1 × 10^6^ leukocytes^9^. Viable CTCs are required for further downstream genotype and phenotype analysis to determine cancer progression. The heterogeneous morphology of cancer cells makes their isolation technically difficult^10^. A cell sorting device with high sensitivity is needed to characterize and isolate viable CTCs. The detection of CTC from whole blood often referred to as “liquid biopsy”, has gained the attention of the scientific and clinical community^11^, because this method has potential for diagnosis, prognosis and evaluation of treatment efficacy^12^. To date, several methods have been devised for the detection, and isolation of CTCs^13–20^. Isolation techniques exploit biophysical properties of CTC which distinguish them from whole blood. Microfluidic cell sorters are most commonly used for diagnostic purposes. Microfluidic technology is used to investigate fluid transport processes in microchannels. Its advantages include small sample size, short reaction time, and low cost. Lab-on-chip (LOC) devices with integrated functions based on the same technology have been developed to carry out biological analysis^21–23^. Microfluidic cell sorters are broadly classified as active or passive. Active techniques use external stimulation like electric, magnetic, optical, acoustic, and biochemical, etc.^22^. In passive techniques no external force is applied , rather, they utilize intrinsic properties like size, shape, channel architecture, and hydrodynamic forces^23^. Some techniques require labeling of CTCs before separation^24,25^. Samples are labeled using certain cell surface markers for example fluorescent labeling, pre-staining, attachment, etc., to enable enumeration and visualization post-separation. The focus of the researchers has been towards the development of CTC enumeration and detection methods including the CellSearch system. Major efforts have been done using immunolabel techniques specific to epithelial cells, for example, EpCAM or various cytokeratins. However, they have a risk of losing the CTCs which don't express EpCAM or have undergone epithelial to mesenchymal transition (EMT)^4,26,27^.

Passive Inertial separation methods offer high throughput, label-free separation with simple and robust designs^28^ thus making them more promising. In inertial microfluidics geometrical changes for example curvilinear geometries and contraction–expansion array (CEA) induce a transverse secondary Dean flow in the microchannels to separate cells based on their physical attributes. This hydrodynamic separation uses massive and high-throughput filtration of blood cells and can accommodate a very high flow rate. Despite the popularity of passive methods, these have certain limitations for example, leukocyte contamination in CTCs due to cell size overlap in case of size-based separation^29^; the design is usually based on sample owing to different geometries for different target cells^30^. Active methods, particularly di-electrophoresis (DEP) uses varying external electric field and intrinsic electrical conductivity of cells to separate CTCs. DEP is an active technique that separates cells based on their size and electrical properties. DEP force refers to the force exerted on the induced dipole moment of a dielectric and/or conductive particle by a non-uniform electric field^31–33^. Normal peripheral blood cells and CTCs of all types of cancers and solid tumors show consistent dielectric differences when exposed to varying electric fields^17,19,34,35^ therefore, DEP can accurately isolate CTCs from a wider range of cells. This isolation does not require any cell surface marking protocols. Since isolated CTCs are unmodified or viable, they can be used for molecular characterization to determine cancer progression, which generally helps in developing more effective personalized therapy. Taking the advantage of the difference in electrical properties among CTC and other blood cells certain DEP-based sorters have been used in the detection of tumor cells of different types such as colorectal, breast, prostate, and lung carcinomas. Most of the DEP-based techniques used binary, spiked samples and not the whole blood^36–39^. Sample preparation for these devices is quite cumbersome and complicated while on other side, handling whole blood is quite challenging as well. Although the DEP technique exhibits accurate CTC isolation however there are certain limitations of like low throughput^40^ and reduced separation efficiency for chip-based DEP microfluidic due to the high conductivity of blood^41^. Additionally, DEP results in heating within the device which leads to a change in the electrical properties of the cell and cell damage^42^.

Considering the individual limitations and advantages of active and passive techniques, hybrid microfluidic devices were proposed. The introduction of multiple forces by combining active and/or passive methods yielded high purity , high sensitivity of separated cells, and an increased operational range of devices while overcoming the limitations of both techniques^22^. In the available open literature, many researchers combined DEP with many active and/or passive methods to improve CTC isolation efficiency. DEP has also been used alongside other active techniques for example magnetophoretic, acoustophoretic, optophoretic. At first, DEP was combined with field flow fractionation (FFF) in which cell equilibrium is achieved using hydrodynamic and DEP forces. Cells are separated based on their densities and dielectric properties and dynamic physical properties of tumor cells were measured^43^.

In an earlier study by Moon and colleagues combined DEP with multi orifice flow fractionation (MOFF) technique to separate MCF-7 breast cancer cells from RBC and WBC at a high flow rate 126 µl/min^39,44^. The sample was prepared using WBC and RBC obtained through centrifugation of whole blood and spiked CTCs. The separation efficiency of CTCs in the reported study was 75.18% due to CTC loss in serial separation and RBC contamination. They presented a rather long configuration (around 10 cm).

Apostream, a device combining DEP with a passive field flow fractionation (FFF) method, used peripheral blood mononuclear cells (PBMNs) isolated from 7.5 ml of normal human blood and spiked tumor cells ovarian cancer SKOV3 and breast cancer MDA-MB-231 cells into it. The reported average recovery of tumor cells from this device was 75.4% ± 3.1%and 71.2% ± 1.6%^11^. Though the separation was improved, the reported device had certain issues like limited throughput and cell overloading causing dipole–dipole interactions, clustering, and mixing of cells.

The DEP-FFF has a limited throughput due to batch-mode processing. A research group introduced continuous flow microfluidic platform which was 160 mm long and 25 mm wide. The sample was prepared using prelabeled MDA-MB-231 breast cancer cells spiked into PBMNs. It has average separation efficiency of 75% and is capable of processing 10 ml samples in less than an hour^45^. However, the large configuration raises the question of whether the same purpose could be achieved in a shorter arrangement. Another research group presented DEP-inertial coupled platform for particle separation. They used polystyrene particles of different sizes in a serpentine channel with vertical negative DEP (n-DEP). DEP-force levitated the particles and their focusing can be done vertically by altering the flow rate and voltage in real-time without the need for redesigning. They reported separation efficiency of 100% and 96% for 13 µm and 5 µm particles respectively^46,47^. An integrated cell separation system was reported using hydrophoresis alongside DEP^48^. In hydrophoresis, hydrodynamic pressure gradients are induced by microstructures to manipulate the particles in suspension. The hydrophoresis module was based on a serpentine channel with microstructures and DEP was applied using a slanted electrode array. System was tested to separate astrocyte-biased cells from mouse neural stem cells with a flow rate of 3.5ul/min^49^. It has been found that cell–cell interaction becomes significant when the hematocrit of blood exceeds 1%^50^. The overloading can be resolved by diluting the blood sample, but only up to 1:10, as beyond this ratio, pH of the blood changes prompting the blood cells to change their biological characteristics (cell membrane)^51^.

The electrical conductivity of blood and its hemoglobin level has a direct relationship^52^. If hemoglobin is separated from the whole blood, it will reduce the cell overloading and joule heating. Therefore, we hypothesize that if RBCs, containing hemoglobin, would be separated from the whole blood, it will reduce the conductivity, cell overloading, and joule heating during DEP separation of the remaining sample. Early separation of RBCs and platelets from the sample will circumvent the cell–cell interaction issue as the hematocrit level will drop. This could reduce the processing time of CTC isolation as well. Moreover, longer exposure to the electric field also causes stress in the cells that can be reduced by pre-enrichment of CTC and WBC from other blood cells like RBCs and platelets. This can be done by adding a preprocessing stage to the DEP sorter on a single platform. Existing techniques utilize centrifugation platform for pre-enrichment before sorting by DEP. However, previous studies reported cell loss due to cytotoxicity caused by spinning^53^. Moreover, delay in centrifugation results in the mixing of blood with gradient medium. High-speed centrifugation stresses the cells thus activating and altering the protein and gene expression of leukocytes^54^. Similarly, magnetic sorting of the blood sample to remove RBCs modulates the surface protein expression of many cancers, especially those originating in the epithelial tissues, making isolation difficult due to changing surface antigens^55,56^. In inertial microfluidics parameters that control secondary flow are Reynolds number Re, Dean number K, and channel’s aspect ratio AR^57^. Thus, various devices of desired efficiency and output can be developed by manipulating these parameters. It is generally accepted that an instrument should complete an analysis of CTCs for a clinically relevant procedure for one sample in two hours^58,59^. We hypothesize that with a combination of these geometries and manipulating the Dean flow, the configuration could be shorter and the separation process would take place in comparatively less time. Due to the scarcity of the CTCs and complications in handling peripheral blood samples, the majority of the researchers use binary or spiked samples to test their proposed devices. Such practice might not be valid when compared with the results of the whole blood sample.

In this research article, all the above-mentioned issues including cell overloading, cell–cell interaction, joule heating, modulation of surface protein expressions, cell loss, and processing time have been addressed using another separation mechanism integrated with DEP in a microfluidic environment. We propose here a novel two-stage serially integrated microfluidic device to separate RBCs and platelets from the whole blood using inertial focusing and then CTCs will be isolated from leukocytes using DEP. The proposed hybrid method takes peripheral blood as input. The device has been designed to reduce cell overloading, increase the throughput and purity as it combines the advantages of more than one method. The addition of another stage eliminates the risk of sample loss and has improved the isolation efficiency compared to existing methods. This study aims to design a microfluidic device for the detection of glioblastoma (GBM) tumor cells. GBM cells inhabit the normal brain region through diffusion and failure in detection results in surgical incurability and the patient succumbs within 14.2 months^60^. In open literature, such a hybrid DEP device for the isolation of GBM cells have not been discussed.

The rest of this paper is organized as follows: Section II explains the concept and design of the proposed device, modeling and simulation details are provided in Section III. The main results,related discussion, and optimization of the design parameters are presented in Section IV. The conclusions are in section V.

## Methods and design

**Figure 1:**
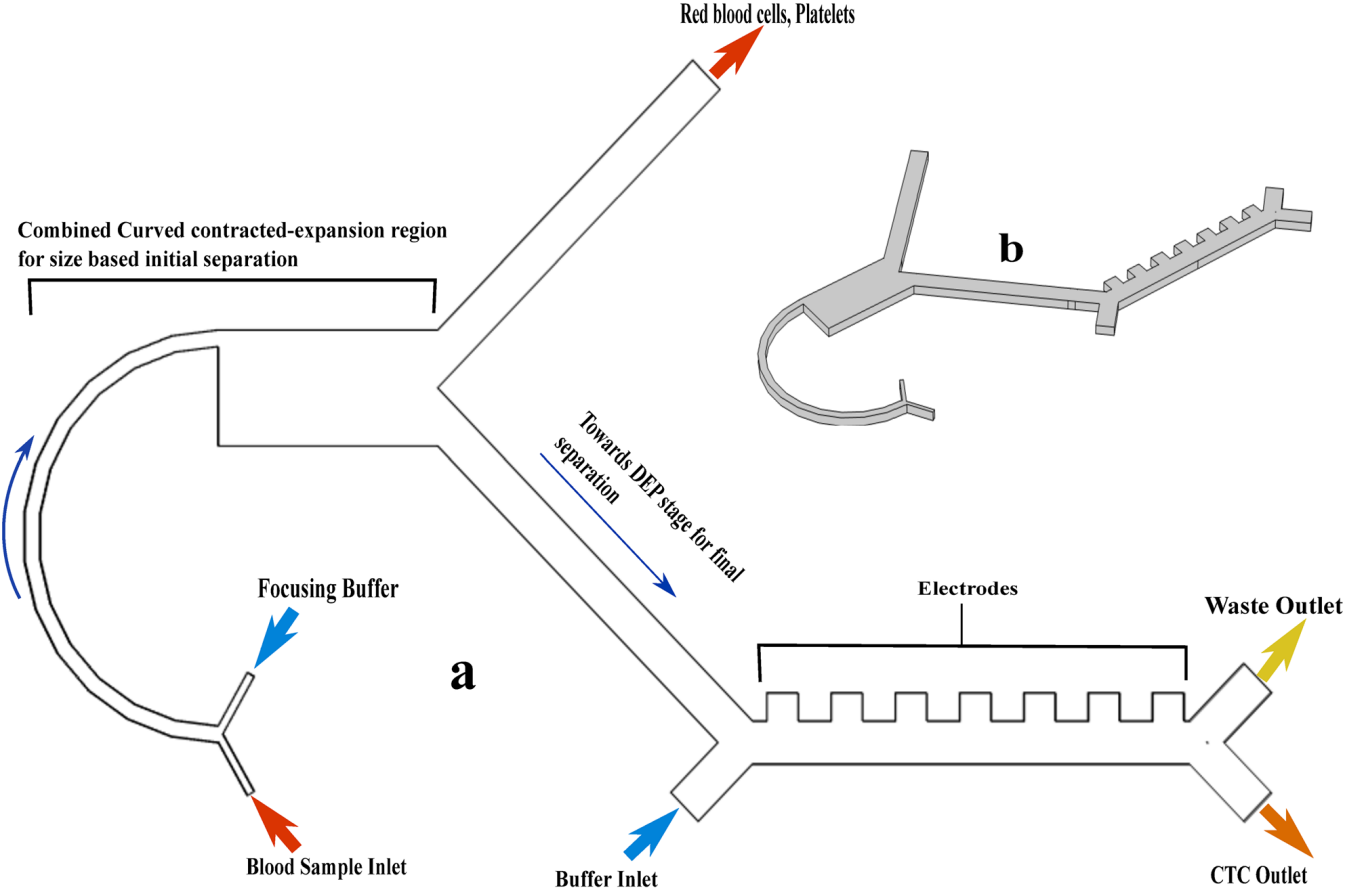
The 3D schematic representation of the developed device with both embedded stages. Once the blood sample is introduced through the inlet, most of the blood cells including RBCs, platelets, T-lymphocytes, and B- lymphocytes are separated in the inertial focusing stage and second stage. Finally, the rare circulating tumor cells are selectively separated using DEP and collected through outlet-II. Residual WBCs exit through waste outlet-III.

The proposed design consists of a serially integrated combined curvilinear inertial focusing channel(passive stage) and DEP sorting channel (active stage). The first stage has two inlets, one for the sample injection and the other for the focusing flow i.e., buffer solution. The injected sample and buffer follow the designed combined geometry to the bifurcation region from where separated CTCs and leukocytes are delivered to the second stage while RBCs and platelets get accumulated in the waste outlet. The inlet of the second stage is symmetrically configured with the outlet of the first stage to maintain the continuous flow field conditions. The second stage selectively isolates hGBM cells via DEP. The second stage comprises two inlets, a set of electrodes, and two outlets. The first inlet of this stage is connected to the outlet of the first stage so that the separated cells become the input of the second stage. Another inlet is for the injection of a buffer. One outlet is for CTC collection and the other one is for WBCs (waste outlet). Figure 1 presents the microfluidic device with an integrated passive stage and active stage.

When the fluid enters into the curved contraction rectangular region, the particles in it experiences inertial lift forces and Dean drag force. The direction and magnitude of the particle migration depends on the size of the particle and balance between these forces**.** Since the channel width is larger than the height, high shear rate is induced between the upper and the lower parts than the side walls. This results in motion of particles towards upper and lower part of the channel due to strong inertial lift forces. Therefore, by changing channel aspect ratio size based inertial separation can be done due to changing shear rate and hence inertial lift force.

The inertial focusing flows are governed by three major forces: a wall induced force, a shear gradient lift force and a secondary flow drag force. A cell flowing through a microchannel will interact with its walls that causes (a) it to move slower than the fluid, and (b) build a pressure between cell and the wall which develops a force directed opposite to the channel wall. This force is inversely proportional to the distance between the particle and the channel wall. The Eq. 1 describes the wall induced lift force^61^ as:

1 $$F_{WL} = v\frac{{r^{4} }}{{D^{2} }}\left[ {\beta^{2} D_{1} \left( s \right) + \beta \gamma D_{2} \left( s \right)} \right]$$

$$\beta = \left| {D\left( {n.\nabla } \right){\text{u}}} \right|$$

$$\gamma = \left| {\frac{1}{2}D^{2} \left( {n.\nabla } \right)^{2} {\text{u}}} \right|$$

$$u = \left( {I - \left( {n \times n} \right)} \right)$$

where *v* is the ratio of fluid density ρ to fluid viscosity µ, *r* is the particle radius, *D* is the distance between the channel walls, *s* is the nondimensionalized distance from the particle to the reference wall, d/D so that 0 < *s* < 1 for particles in the channel, *D*_1_ and *D*_2_ are functions of nondimensionalized wall distance *s,* n is the wall normal at the nearest point on the reference wall, I is the identity matrix and v is the fluid velocity.

As the typical microfluidic velocity profile is parabolic, therefore a cell will be exposed to velocities of different magnitudes simultaneously which causes the fluid to induce a force on the cell in the direction of increasing shear i.e., channel wall. This force is presented in Eq. 2.

2 $$F_{SG} = C\rho {\text{v}}_{{\text{m}}}^{2} r^{6} {/}D_{h} ,$$

where C is constant, $D_{h} = \frac{2wh}{{w + h}}$ is the hydraulic diameter (w is width of the expansion region and h is height of microfluidic channel), v_*m*_ is the maximum channel velocity. This equation shows that this force depends on channels Reynolds number and particle position but is independent of particle rotation. This shear gradient lift force used in simulation is provided by^62^. Assuming particle Reynolds Number < 1 and parabolic flow, the total magnitude of these lift forces is provided by Evgeny^63^

3 $$F_{L} = \rho G^{2} r^{4} f_{L} ,$$

where $G$ is local shear rate and $f_{L}$ is defined as lift coefficient. This function depends on the particle position in the channel and the channel Reynolds number. Regarding secondary flows, they are used to control the number of equilibrium positions within a given channel crosssection. Equation 4 shows the drag force applied by these secondary flows. Assuming Stokes drag, it is given by^64^.

4 $$F_{D} \sim U_{m}^{2} r^{2} D_{h} \rho {/}R$$

where R is the radius of curvature of the curved channel. This drag force is directly proportional to the secondary flow velocity and the cell size. However, it is inversely related to the radius of curvature of the curved region of the channel and channel Reynolds number.

The proposed design utilizes curved and CEA, and therefore the forces vary as the geometry changes. The balance between these two forces determines the final equilibrium positions of the particles. The particles tend to locate between the channel wall and center under the effect of inertial lift. Equation 3 shows that the force scales linearly with the cell radius. This causes large particles to move towards the channel wall .Once there, these cells remain unaffected by the dean drag force and maintain a distance from the vertical center of the channel. Smaller cells are not affected by the inertial lift force and thus move toward the center under the effect of radially directed dean flow. Thus, cells are separated according to their size at the channel outlet.

The ratio of the accumulative lift and drag forces $\frac{{F_{L} }}{{F_{D} }}$ is the determining factor as to where a cell of a given diameter equilibrates in the channel by identifying that which force dominates. When the ratio is >  > 1, inertial focusing of microparticles happens while when it become <  < 1 the dean drag force mixes particles^65,66^. Equation 3 shows that inertial migration intensity depends on system parameters and that it is directly propotional to two times the cell size. Therefore, cells with large diameters tend to move toward the channel wall and achieve an equilibrium position somewhere between the wall and center. The cell/particle focusing by dominant inertial lift force is strongly dependent on the ratio *a*_p_ /*D*_h_ where a_p_ is the cell diameter^64,67^. In this ratio hydraulic diameter is an important factor due to change in channel height at the same condition of Reynold’s number. According to our proposed approach and hypothesis, prior efficient separation of red blood cells and platelets (size ~ 2-4 µm) from the whole blood could facilitate the CTC isolation and recovery in DEP stage. From the findings of Lee et al.^68^, a CEA microchannel with low aspect ratio enable the separation of particles under 4 µm in diameter high flow rate and throughput. Therefore, we opted low aspect ratio of the contraction region of the inertial microfluidic channel. Considering the diameters of all the target cells, we initially designed different Curved-CEA microfluidic channels with low aspect ratio (AR = H/W) and channel height ranging from 40-100µ. We studied the behaviour of cell focusing by quantitatively exploring the effect of the modulation of the inertial force. The *a*_p_ /*D*_h_ ratio of CTCs at an aspect ratio of 0.8–2 were calculated with 0.17–0.07, respectively.

After separating from the rest of the blood cells, CTCs with residual blood cells (mostly monocytes and granulocytes) proceed to the second stage where an active technique is applied to isolate CTCs from leukocytes accurately. DEP force refers to the force exerted on the induced dipole moment of a dielectric and/or conductive particle by a nonuniform electric field^69^. The force acts only if the differential between particle’s permittivity and that of the surrounding fluid exists. The intensity of the dielectrophoretic force on the particles depends on the permittivity gradient between the suspension and the particles. If a biological homogenous spherical cell of radius r_p_ is placed in a varying electric field while being floating in a medium, the total dielectrophoretic force, neglecting higher order of polarization, can be estimated as^70^

5 $$F_{DEP} = 2\pi r^{3} \varepsilon_{0} R_{e} \left( {CM} \right)\nabla \left| {E_{rms} } \right|^{2}$$

where ε_0_ = 8.854187817 × 10^−12^ F/m is the permittivity of vacuum, Re(CM) is the real part of Clausius–Mossotti factor describes the frequency variation of the effective polarizability of the particle in the medium^44,69^. It depends on complex permitivities of the cells, suspending medium and frequency of the externally applied electric field^70,71^. Since it is a function of electric field frequency, it plays a significant role in the separation process. Biological cells are not homogenous due to the lipid bilayer cell membrane and intracellular structures and fluid, therefore, to calculate CM factor, we considered a single shell model presented by^70^ assuming a cell as a particle with a thin outer layer (shell) or cell membrane. The volume inside the thin layers or intracellular structures and fluid is assumed to have uniform conductivity and permittivity, but the properties of the cell membrane can differ significantly from those of the rest of the cell. In single shell model, while computing DEP, the equivalent complex relative permittivity of a homogeneous particle comprising both the cell membrane and cytoplasm of the cell is applied instead of the complex permittivity of the particle. If the complex permittivity of cells becomes greater than that of suspension fluid $\varepsilon_{p}^{*} > \varepsilon_{f}^{*}$, it is termed as positive DEP and it attracts the cells towards the electrodes. In contrast, negative DEP repels the cells or particles from the region of a high electric field.

**Figure 2:**
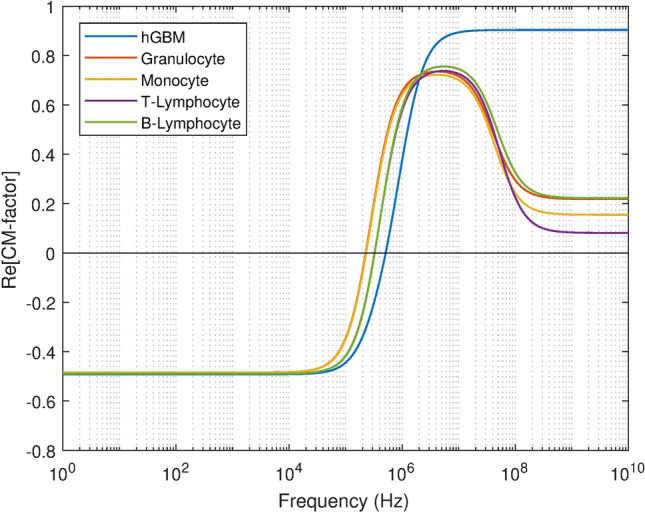
Real part of Claussius–Mossotti (CM) factor of hGBM and subtypes of leukocytes is mapped against applied frequency using single shell model.

In this study, negative DEP repulsion is used in combination with hydrodynamic focusing. The separating mechanism in case of DEP separation is that to find an AC frequency, where the CTCs experience nDEP, while the remaining other leukocytes undergo either positive DEP or no DEP(cross over frequency). The DEP force acting on the cells is proportional to their cell volume. The total force $F_{T} = { }F_{DEP} - f\nu ,$ acting on the cells is given by dielectrophoretic force, opposed by the hydrodynamic drag force. Where *f* is the Stokes drag force and for small Reynolds numbers it is given by *f* = 6πηr, ν is the flow velocity of cell-suspension. Since cells are treated as rigid spherical objects in a medium of viscosity η, the velocity due to DEP is hence proportional to the square of the cell radius. The particle’s trajectory is estimated after computing the forces in the flow field. The motion of cells in a fluid follows Newton’s second law of motion^72^. The net force on a cell in DEP stage is given as:

6 $$F_{T} = m_{p} \frac{{d{\text{v}}}}{dt} = F_{L} + F_{D} + F_{dp} + F_{vm}$$

where *m*_p_ is the cell mass, ${\text{v}} = d{\text{q}}/dt$ is the cell velocity, and q is the position of the cell, *F*_*L*_*, F*_*D*_*, F*_*dp*_ and *F*_*vm*_ are the lift, drag, pressure gradient, and virtual mass forces, respectively. The CM factor specifies the sign of DEP force based on the working frequency. Figure 2 illustrates the CM factor using a single shell model for hGBM and IV types of leukocytes versus applied frequency. The intensity of the dielectrophoretic force on the particles depends on the permittivity gradient between the suspension and the particles. The DEP stage consists of a set of interdigitated electrodes along the main channel for cell suspension and two outlets for CTCs and other is the waste channel for leukocytes. Appropritate voltage and frequency are selected such that when the CTC approaches the electrodes, it experiences nDEP force, causing it to move away from the area of high electric field and eventually to the outlet. While, the leukocytes will undergo pDEP and move towards the other outlet. As most of the content of blood (Non-Newtonian) has been removed in the first stage therefore, 0.1 × Phosphate buffer saline (PBS) of low conductivity 0.055 S/m having relative permittivity 78 was selected for 2nd stage. The velocity of the particles was reduced due to an increase in channel width from 50 to 175 µm. This reduction in velocity allowed all the cells to become exposed to the DEP force. It also decreased the electric field across the channel cross-section, which resulted in viable cell retrieval making the cell isolation easier at the outlets.

### Computational modeling

COMSOL Multiphysics v5.5 is used for modeling the device and a finite analysis module was used to conduct the computational fluid dynamics (CFD) simulation. COMSOL provides an integrated graphical user interface (GUI) where models can be built and solved by using predefined physics modules optimized for specific application areas. We used fluid flow, micro-electromechanical system (MEMS), and electric circuits modules. The objective of 2D- and 3D modeling was to compute the flow field, validate the forces and visualize the separation process within the proposed device. CAD model of geometry was developed using the built-in geometry feature of COMSOL.

The proposed design of the curved-CEA inertial separation stage is inspired from the work of Shamloo et al. The initial width of the curved region is 50 μm and length is 1950 μm while the width of the expansion region is 350 μm and length 700 μm. Channel height is 50 μm.

After geometry design, the next step was to define the domain-wise material of the model. Though COMSOL is complemented by a huge material library, to develop a realistic device design that could be used for clinical testing, the physical parameters of human blood sample associated with both separation stages were extracted from the literature^73,76–78^ and their values are in Table 1. This study focused on brain tumor cells or human glioblastoma cells (hGBM) for which no previous DEP separation has been reported in the open literature.

**Table 1:** Physical and electrical properties of cells used in the proposed technique. These parameters were directly accounted for in our model and resulting computation.

Cell type	Mean diameter (μm)	Cytoplasm permittivity	Cytoplasm conductivity (σ = S/m)	Membrane permittivity	Membrane conductivity (σ = S/m)	Membrane thickness (nm)	Literature
hGBM	12.2	1.00E + 04	0.25	5	1 × 10^−6^	10	^73,74^
WBC	7.5	150.9	0.76	5	1 × 10^−6^	4	^74,75^
Granulocyte	9.42 ± 0.46	150.9 ± 39.3	0.6 ± 0.13	5	1 × 10^−6^	4	^76^
T-Lymphocyte	6.58 ± 0.7	103.9 ± 24.5	0.65 ± 0.15	5	1 × 10^−6^	4	^77^
B-Lymphocyte	6.58 ± 0.52	154.4 ± 39.9	0.73 ± 0.18	5	1 × 10^−6^	4	^77^
Monocyte	9.26 ± 0.72	126.8 ± 35.2	0.56 ± 0.1	5	1 × 10^−6^	4	^77^

**Figure 3:**
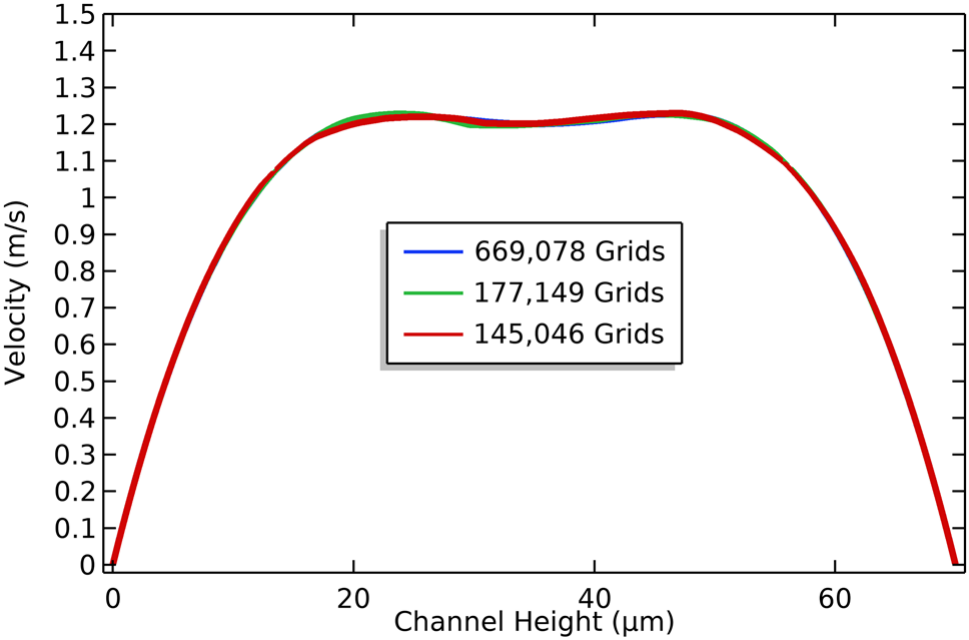
Velocity distribution (y-component) along the vertical line drawn at the center of the expanded region shows the velocity variation in the specified region of the microfluidic channel. Mesh convergence analysis was done with 145,046, 177,149 and 669,078 elements or grids i.e., (coarser, normal, and finer mesh settings). The line plot indicates the mesh convergence for the 1.28 µm grid size.

In the COMSOL model builder, flow simulation properties were defined in the microfluidic domain. To avoid an accumulation of cells around a sharp cornered area which happened due to multiple areas of zero velocity, curved edges were used. As pointy edges and sharp corners were not optimal for cell separation efficiency^75^. Careful meshing was done to avoid errors like inverted mesh elements. Very fine meshing was done around the sharp and curved regions of the channel. To discretize the computational domain of the proposed geometry, a uniform tetrahedral structured mesh was used. A structured mesh was used for discretizing the geometry of the inertial stage. Mesh Independency analysis was done, with 145,046, 177,149, and 669,078 grids or elements (Fig. 3). Satisfactory convergence of results was obtained with elements sizing between 1.28 for finer and 11.9 μm for coarser mesh. The maximum error rate related to the given number of elements was < 1%.

The finite element method (FEM) was implemented using fluid flow module. The results obtained from the laminar flow simulation were used as input for the particle tracing module. Which in turn generated the particle trajectory in the designed microfluidic domain. Subsequently, the governing PDEs were solved using the simulation software. Navier–Stokes equation and momentum (nonlinear convection–diffusion) equations were solved for the flow field distribution. For low aspect ratio, a_p_/D_h_ is considered to be < 1, therefore flow rate was chosen to be between 120-200 µl/min. The two inlets have been defined by flow rates of 20 and 200 μl/min for a blood sample and focusing flow, respectively. A zero-pressure boundary condition was applied on the outlets of the microfluidic channeland no-slip boundary condition was used for walls. The Galerkin least squares formulation was used to approximate the governing nonlinear PDEs with a system of ordinary differential equations (ODEs). Laminar flow with discretization (P2 + P2) was solved for the fluid flow. It results in the net inertial lift and drag forces along with the virtual mass and pressure gradient force to be used in the particle tracing module to estimate the motion of the cell. For the blood sample to be injected at the inlet, a group of cells involving seven cell lines: 24 RBCs, 12 Plts, 12 WBCs (granulocytes, monocytes, T-lymphocytes, B-lymphocytes), and 3 CTCs is selected. Above mentioned cell (scaled down) count was based on the actual quantity of the same cells in the human blood. Time dependent study with 0.0001 s time step was performed in the particle tracing module. The equations of motion were solved considering stokes drag force with wall corrections, lift force including wall induced and shear gradient, virtual mass, and pressure gradient force.

**Figure 4:**
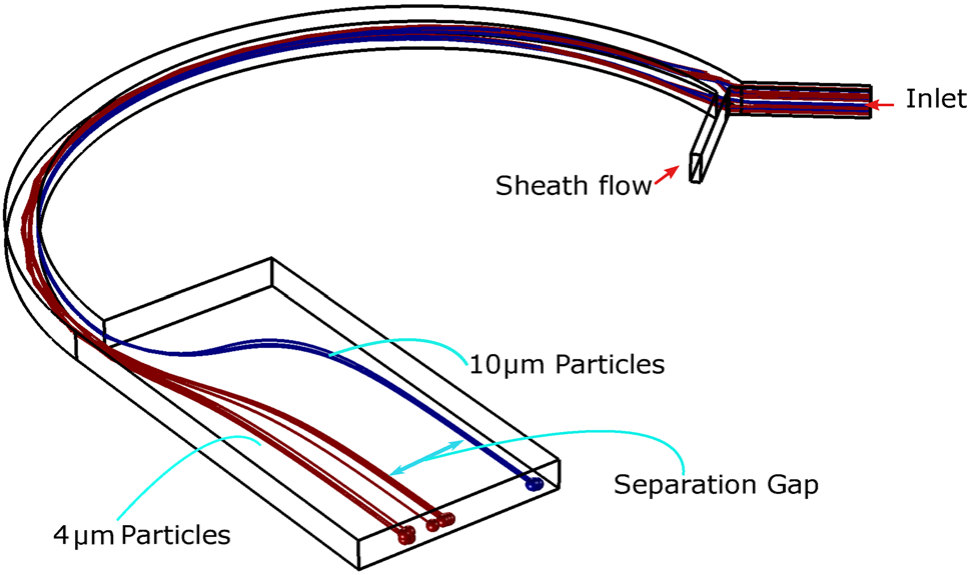
Simulation results on the primary geometry with a single contracted curved and expanded low aspect ratio AR architecture. Model Particles with sizes 4 and 10 µm were injected with the sheath flow. Simulation shows separation of the particles with visible separation gap.

**Figure 5:**
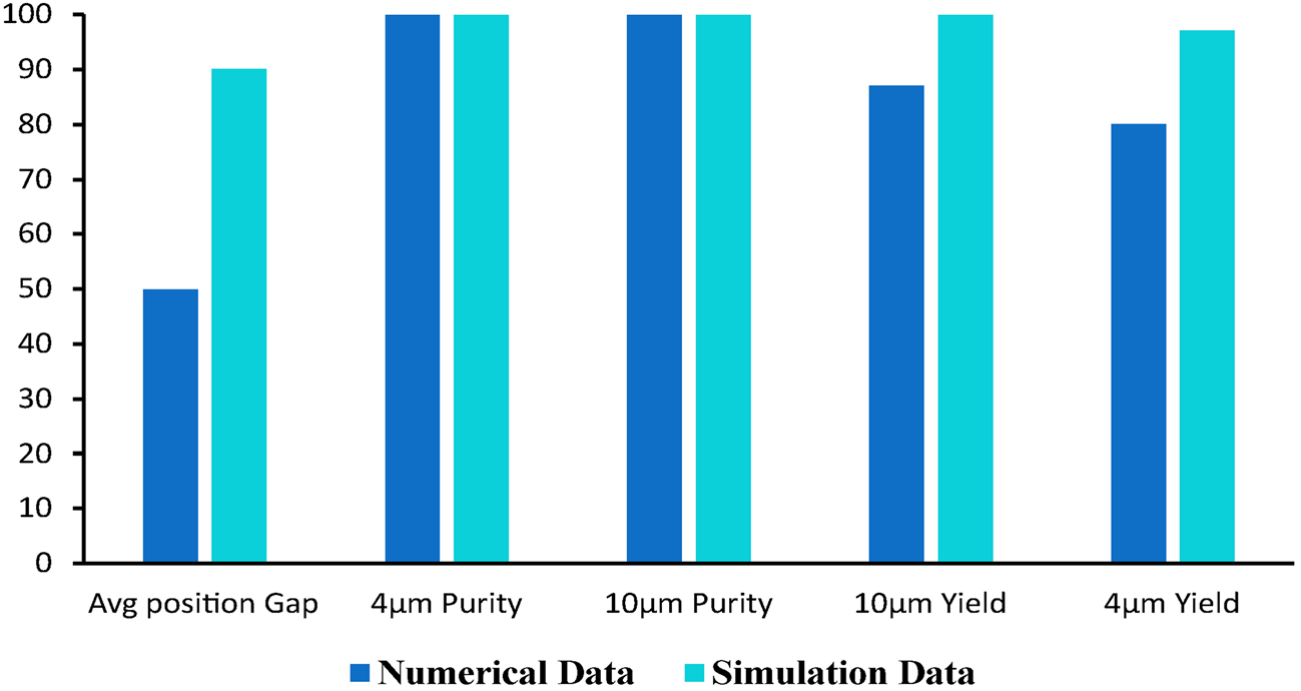
Comparison of primary geometry simulation results and data available in literature. The average separation gap between 4 and 10 µm particles and yield was found to be more in the performed simulation. However, purity of both cell sizes was similar to the reported data.

The primary geometry, used for model comparison in this study, consists of a curved region and one expansion region. The initial width of the curved region is 50 μm and length is 1950 μm while the width of expansion region is 350 μm and length 700 μm. Channel height is 50 μm. For the contraction zone, an aspect ratio (AR = H/W) of 1 is selected. This quantity has been chosen carefully since it is a critical for proper separation. First, the simulation was done using only two model particles of size 4 and 10 µm on the primary geometry **(**Fig. 4) and the results were compared with the results reported by Shamloo et al.^79^ elaborated in Fig. 5. The separation efficiency can be determined as the ratio of CTC obtained in the CTC outlet to the total amount of CTC injected at the inlet. The simulated model describes the position of the maximum cell concentration with a separation gap. To visualize the cell separation, particles were colored based on their sizes in Fig. 4.

**Figure 6:**
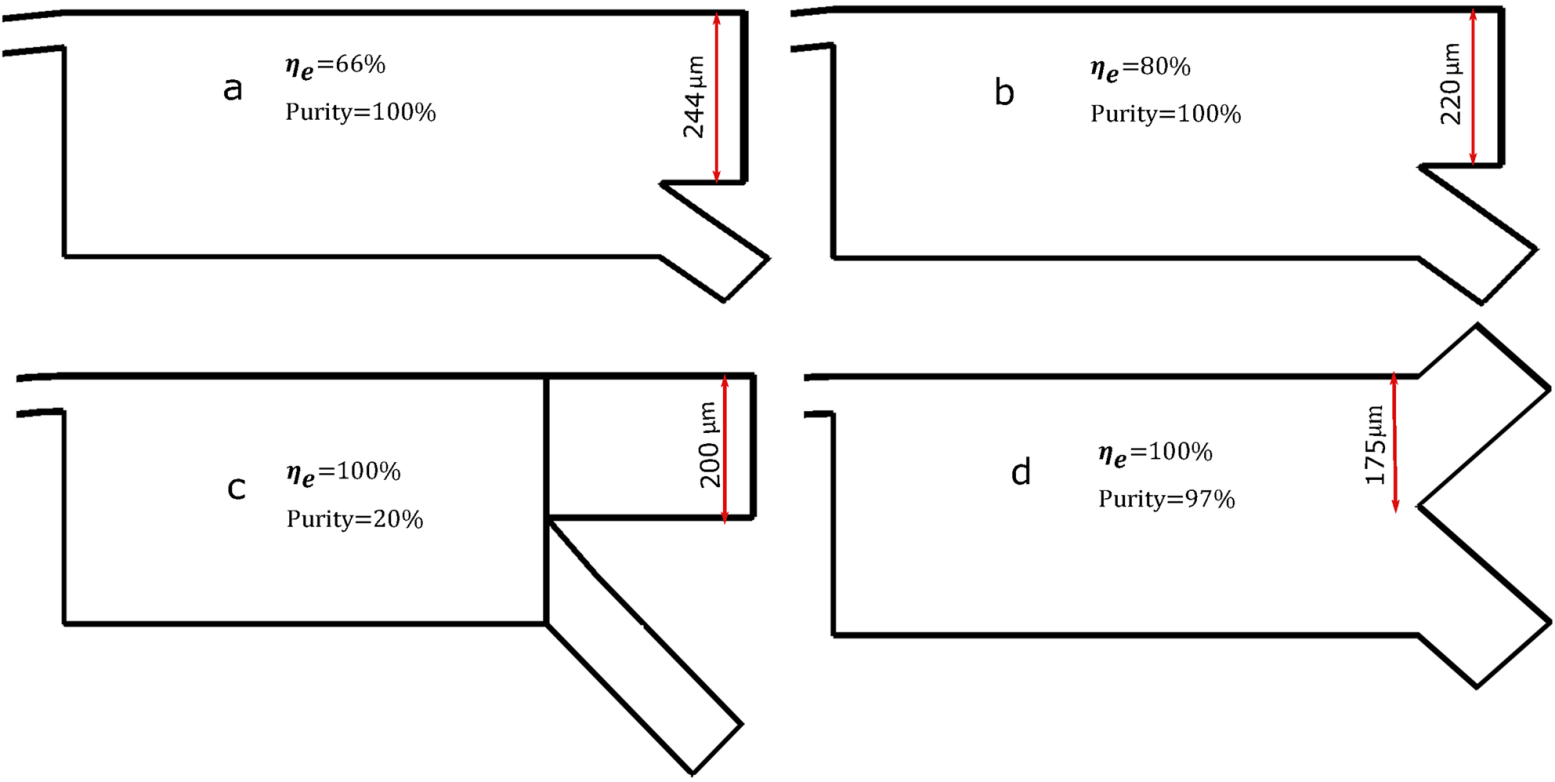
Different designed outlets highlight the selection of inertial focusing phase. To visualize the separation efficiency particles are colored according to their sizes. (**a**) Outlets are designed based on the concentration gap of particles in the initial model. (**b**) Outlet sizes altered (**c**) Waste outlet size increased (**d**) Symmetric bifurcation of the outlets results in the effective separation of CTC from rest of the blood cells.

The comparison reasonable but due to the different cell sizes under study and serial integration of DEP stage, same design didn’t work for hGBM separation. Thus, after establishing the analysis procedure of inertial focusing stage, customized design for RBC and Plt separation from WBCs and CTCs is finalized by incorporating geometrical changes.Bifurcation outlets of different sizes and shapes were designed and tested for separation and collection of the RBCs, Plts, WBC, and CTC. The initial design of outlets was made based on the separation gap between the target cells. centrifugal effect and Zweifach-Fung bifurcation law^80^. Figure 6a–d shows different designs of outlets indicating the change that occurred in the separation efficiency and purity. The design displayed in (d), demonstrated the best separation strength while validating the previous results. Therefore, bifurcation design (d) has been selected for the inertial focusing phase, top outlet is for rejected RBC and Plt while the other one is used for transporting CTC and WBC for further separation. Once design was finalized, several simulations were done to determine separation efficiency and purity i.e., the ratio of the number of cells collected in the desired outlet to the total number of cells in that outlet.at different aspect ratios.

We observed that the cells start focusing as inertial force dominates over the dean drag force around *a*_p_ /*D*_h_ ~ 0.1. From this we can predict that CTC separation from other blood cells can be done at the designed curved-CEA geometry under 70 μm height with Reynolds number Re above18.

For the DEP separation stage simulation electric current, fluid flow, and particle tracing modules were used. The model consists of stationary steady state, frequency domain, and transient study. Velocity, pressure, and electric AC potential distribution fields were calculated using a stationary study, while the transient study was used to generate particles trajectories influenced by DEP force. For the electric-currents physics, the boundary condition of electric insulation was selected for the walls of the microchannel. Defining electric potential on electrodes would override the places of electrodes within the walls. Square shaped electrodes with the sides of 80 μm, were used which are feasible for fabrication. To generate an electric field, electrodes with the same voltage and alternating signs were used. Electric potential ranging from 3.5-5 V was applied on electrodes. To compute electric field, the fine mesh has opted while coarse mesh was generated for solving fluid flow field. Different meshes were used near sharp corners to refine the solution. For the stationary study of fluid flow, an algebraic multigrid solver (AMG) or preconditioner was used as the linear solver, the electric field was computed using iterative solver BiCGStab, and GMRES iterative solver with Jacobi method was used for cell tracking. In the case of particle tracing, the boundary condition for channel walls of DEP stage was set to “bounce” and outlets condition was set to “stick”, which means cells would bounce back or stick to outlets in case of collision, respectively. The separated cells (CTCs and WBCs) from the outlet of the first stage enters into the domain of the second stage. Since most of the volume is rejected along with the RBCs and platelets in inertial stage, more buffer(0.1xPBS) was needed for the focusing flow. The fluid suspension was a Newtonian flow over all in this stage therefore, having a low electrical conductivity ~ 0.055 S/m. Such configuration generates both pDEP and nDEP force to move cells towards or away from electrodes, respectively. From the path of the leukocytes and CTC, the former was spotted close to the electrodes. Therefore, it can be concluded that pDEP force is imposed on WBCs while CTCs experience nDEP force.

## Results

In this paper initially an inertial microfluidic channel was numerically simulated based on the study of Shamloo et al. The device design has been changed according to the desired sample i.e., whole blood after verifying the basic geometry in light of the investigation results of Shamloo et al.^79^ Particles that have been examined were 12.2, 6.58,9.26,9.42, 4 and 2 µm in diameter representing CTCs, WBCs (four major types), RBCs and Platelets respectively. To find the optimal design parameters the relation between channel’s aspect ratio, flow rate and separation efficiency was studied. Afterwards, DEP stage was separately designed and then serially integrated with the inertial microchannel to facilitate and improve CTC isolation from the whole blood sample.

**Figure 7:**
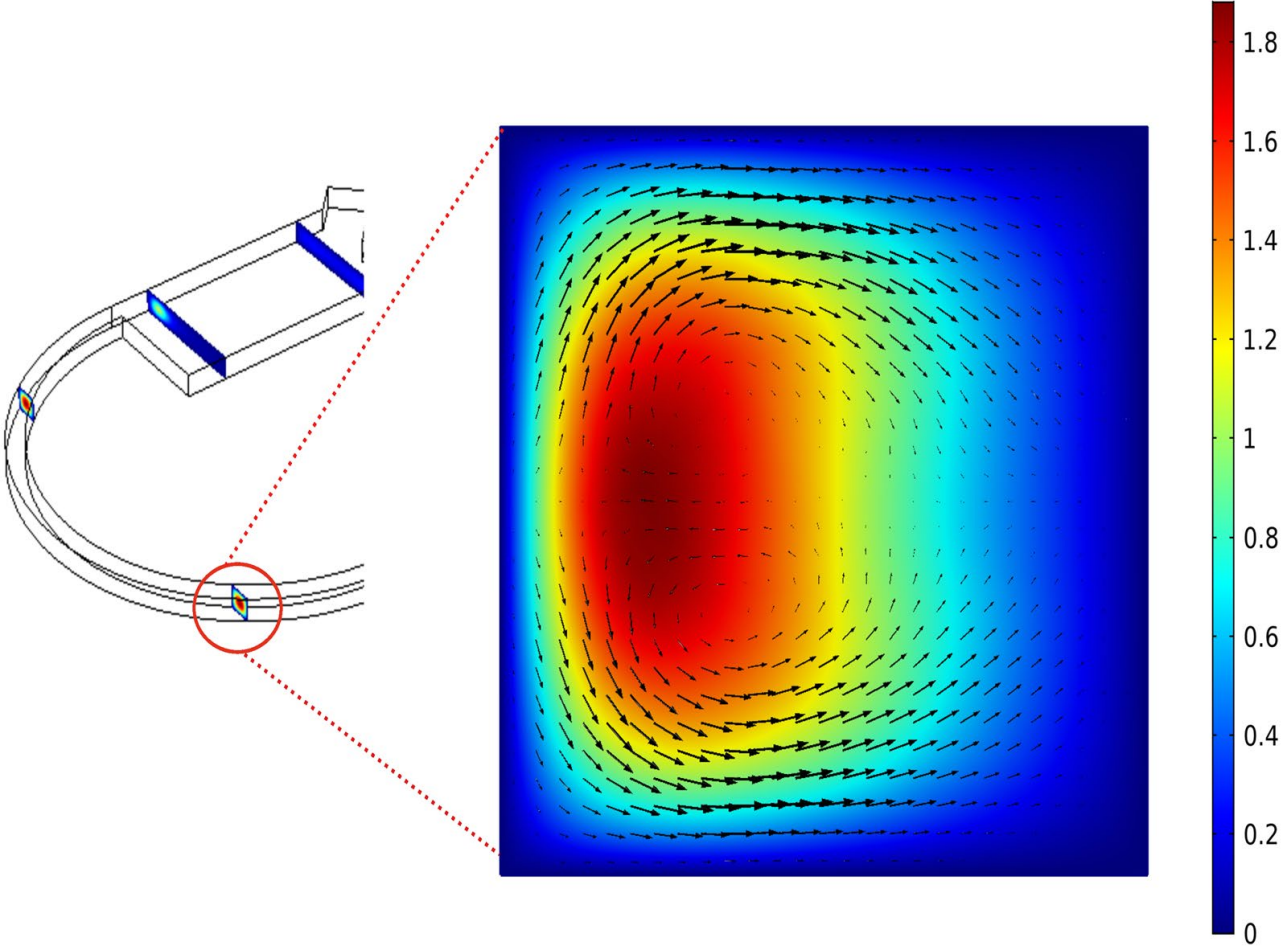
Secondary flow vortex or Dean flow vortices appear in the curved channel and change their strength along the width to achieve maximum velocity magnitude, The arrows show the Dean drag force. Balance between drag and inertial lift forces determines the final direction of the particle across the channel depending upon the particle size. The smaller particles follow the flow streamline and move towards upper outlet while the large sized particles move towards the lower outlet.

The proposed model takes advantage of the curved and contraction–expansion array (CEA) separation geometries. Additional inertial forces are introduced when the microchannel is curved or its aspect ratio changes, (e.g., curvilinear geometries and CEAs, respectively). Such geometrical changes induce a transverse secondary Dean flow in the microchannel. The combined architecture has been designed to strengthen Dean flow vortices to separation level in a shorter distinctive length in comparison with straight CEA geometries. Initially, only four cell lines were considered i.e., RBC, Plt, WBC, and hGBM. Two dean vortices generated within the channel (Fig. 7) reinforce each other resulting in the maximum velocity magnitude for obtaining maximum separation efficiency and purity for CTCs.

**Figure 8:**
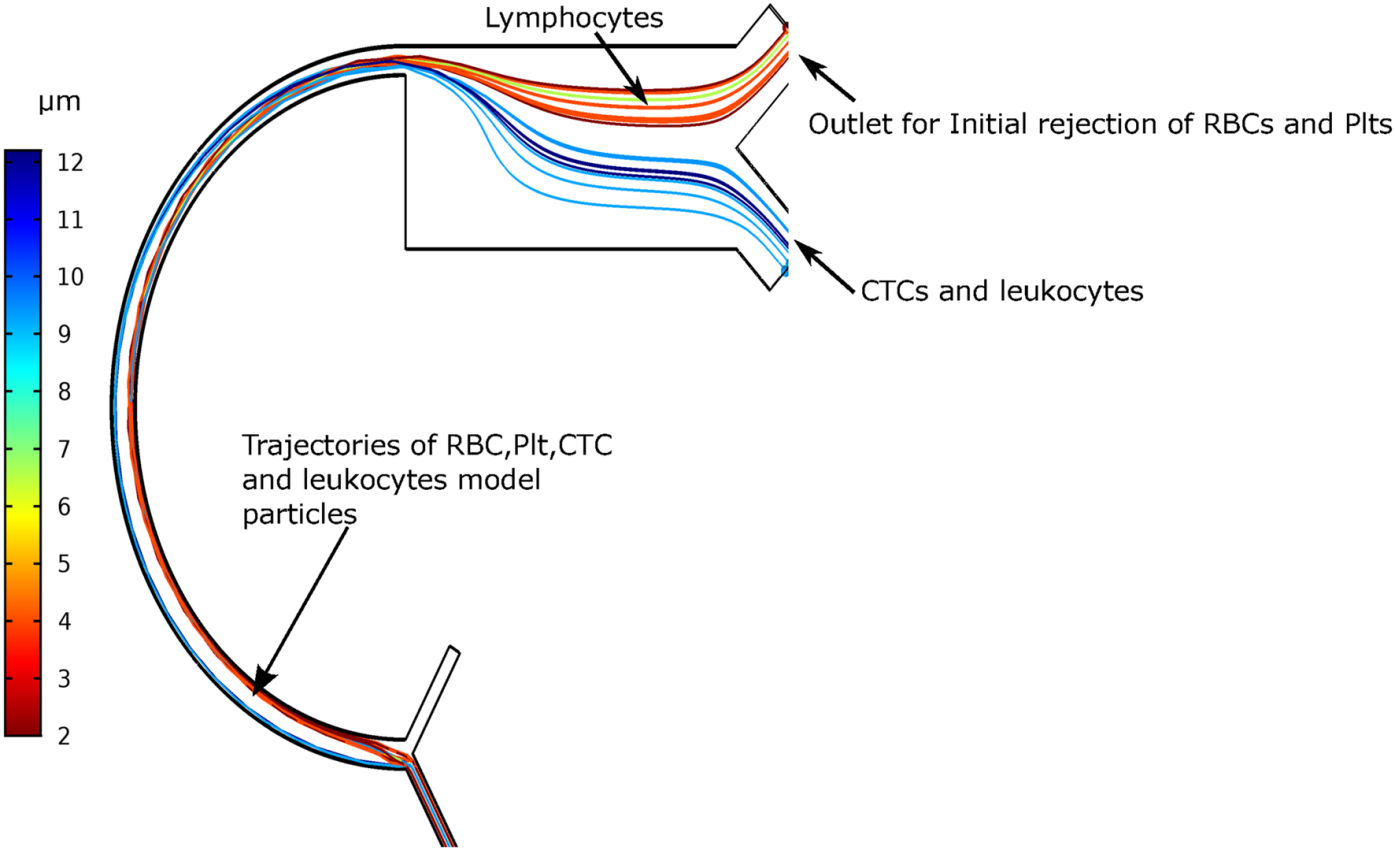
Results of the particle tracing of whole blood sample while considering subtypes of leukocytes. The color legend shows sizes of particles being injected. In the expansion region separation of small particles i.e., RBC and Platelets takes places from the larger particles i.e., CTCs and WBCs and they continue to move towards their respective outlets. However, it has been observed that certain subtypes of WBCs made their way along with RBCs and Plts towards the waste outlet for the initial rejection of RBCs and Plts.

Most of the available literature consider the mean diameter of WBC and neglect the actual variants of WBCs or leukocytes. This adds an inherent error or limitation in the results. Considering practical conditions, we added subtypes of WBCs in our model i.e., monocytes, T-lymphocytes, B-lymphocytes, and granulocytes. Mechanical, and dielectric properties have been exploited to separate WBCs (granulocytes and monocyte) from CTCs. Our analysis show that only CTCs are concentrated towards the right side of the channel, Fig. 8, whereas other cells accumulated on the left. As previously discussed, large cells i.e., CTCs are influenced by the inertial lift force, which retains them at a nearly constant position with respect to the channel wall. On the contrary, the Dean flow created in the curved region leads the rest of the smaller cells to concentrate toward the outlet#2 of stage 1. Figure 8 show the cells’ trajectories from the particle tracing module, interestingly T and B-lymphocytes (6.58 μm) got separated along with RBCs and Plts while granulocytes and monocytes are comparable in size with CTCs were delivered to the DEP stage along with CTCs. It was observed that leukocytes having sizes close to Plts and RBC (2-6 µm) are drained out along with them. While certain types of leukocytes having diameters close to CTC (9–12.2 µm) get along with each other, making their way to the second stage of the microfluidic device. These results have validated our hypothesis that combined geometry with curved and CEA could get the required results with smaller lengths.

**Figure 9:**
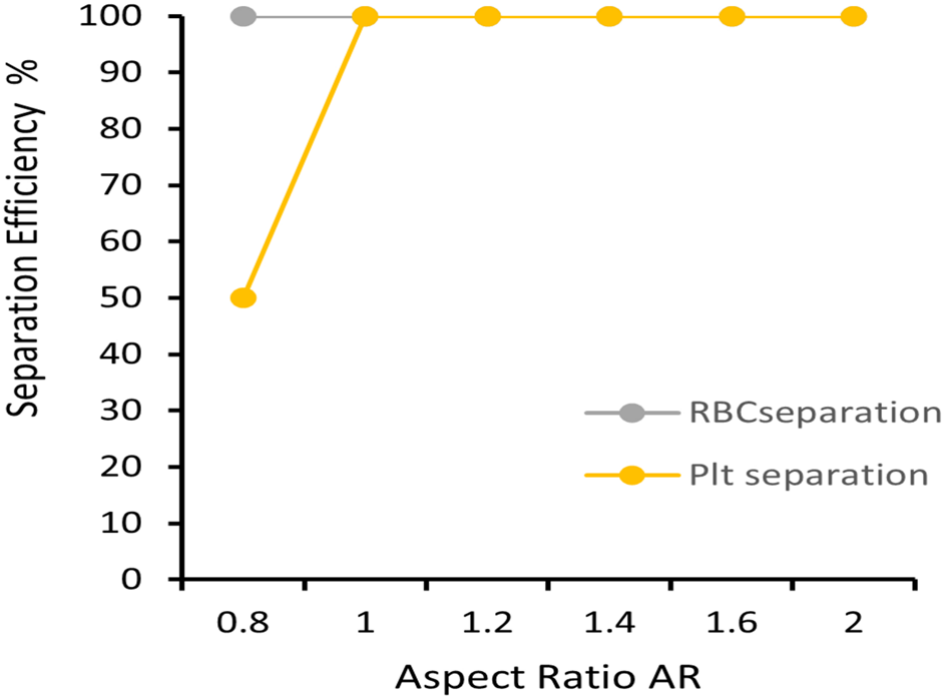
Separation efficiency versus aspect ratio of RBC and Platelets. Low AR of the proposed combined geometry was found suitable for maximum separation efficiency.

To evaluate the performance of the proposed inertial stage we calculated the separation efficiency and purity of the cell suspension at different aspect ratios ranging from 0.8–2. Generally, it was observed that the separation efficiency is poor when the aspect ratio is < 1. At an aspect ratio of 1, separation efficiency of first stage raises to 100% (Fig. 9). Between aspect ratio 1–2, certain types of WBCs whose sizes are comparable to that of RBCs also migrated to outlet#1. Overall, the RBCs & platelet separation efficiency of 100% is obtained for aspect ratio ≥ 1. The threshold of the ratio a_p_/D_h_ is observed as ~ 0.1 for maximum separation efficiency as reported by^81^.

**Figure 10:**
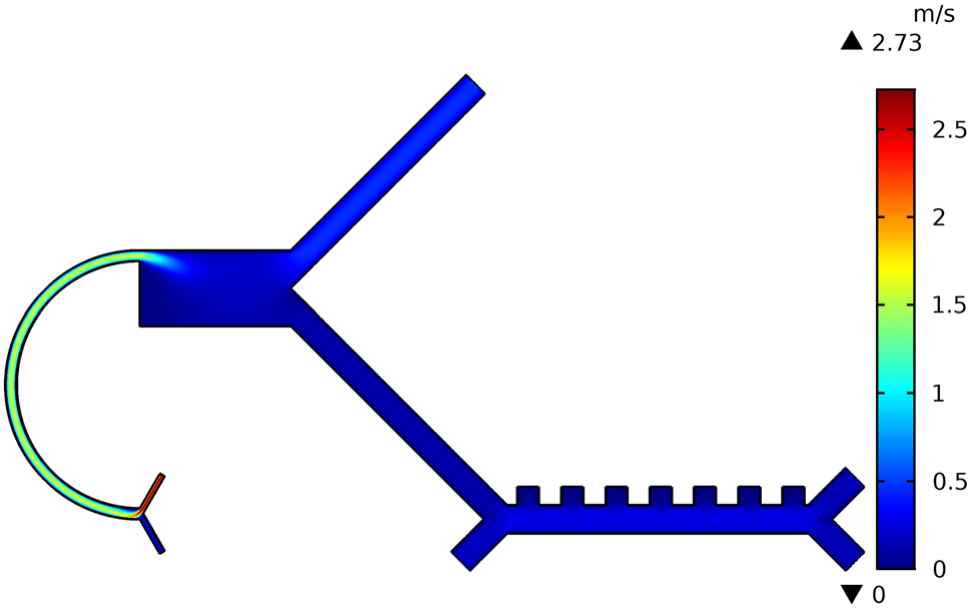
Velocity field distribution of the proposed hybrid device. The surface plot of the velocity indicates the velocity field within proposed device in color spectrum. When the focusing buffer is injected, the velocity around its inlet is high (represented by red region). The cell suspension was injected with a low velocity, thus lowering the fluid stream velocity in the curved contracted region (represented by green region). As the fluid enters in the expanded region it further reduces the velocity (represented by blue region). At the end of the inertial stage, the single channel is bifurcated, hence dividing, and then reducing the volumetric flow. DEP stage channel size is further increased. This equalization of hydraulic resistance promotes the uniform distribution of flow across the DEP stage, where an evenly blue spectrum is obtained with fluid flow simulation.

The velocity field distribution of the two*-*stage microfluidic device is depicted in Fig. 10. The surface plot shows a decrease in velocity as fluid was transferred to the expansion region and then to the symmetrical bifurcated downstream channels. This happens due to hydraulic resistance, which directly links to the dynamic pressure of the fluid. The velocity magnitude changes due to the change in the strength of the secondary flow vortex and due to geometrical changes through the channel. The velocity of the particles was reduced due to an increase in channel width from 50 to 175 μm. This reduction in velocity subsequently slowed down the cells and allowed them to become exposed to the DEP force. It also decreased the electric field within the channel, which resulted in viable cell retrieval making the cell isolation easier at the outlets.

Figure 1 shows the proposed design of the DEP stage, to ensure continuous cell separation with fluid flow, dynamic pressure, and velocity generated within the designed channel were analyzed. For the effective continuity of the flow, the dynamic fluid pressure of the microfluidic channel was examined. DEP stage is governed by parameters including applied voltage, frequency, and the number of electrodes. The CM factor of leukocytes and hGBM is calculated versus the applied frequency in Fig. 2. The crossover frequencies of leukocytes i.e., monocytes,granulocytes, T-lymphocytes and B-lymphocytes ranges from 220-330 kHz. However, crossover frequency of hGBM is around 511 kHz. Therefore to isolate CTCs from leukocytes, frequency ranging between 331-500 kHz may be used for inducing pDEP to leukocytes and nDEP to the hGBM.

**Figure 11:**
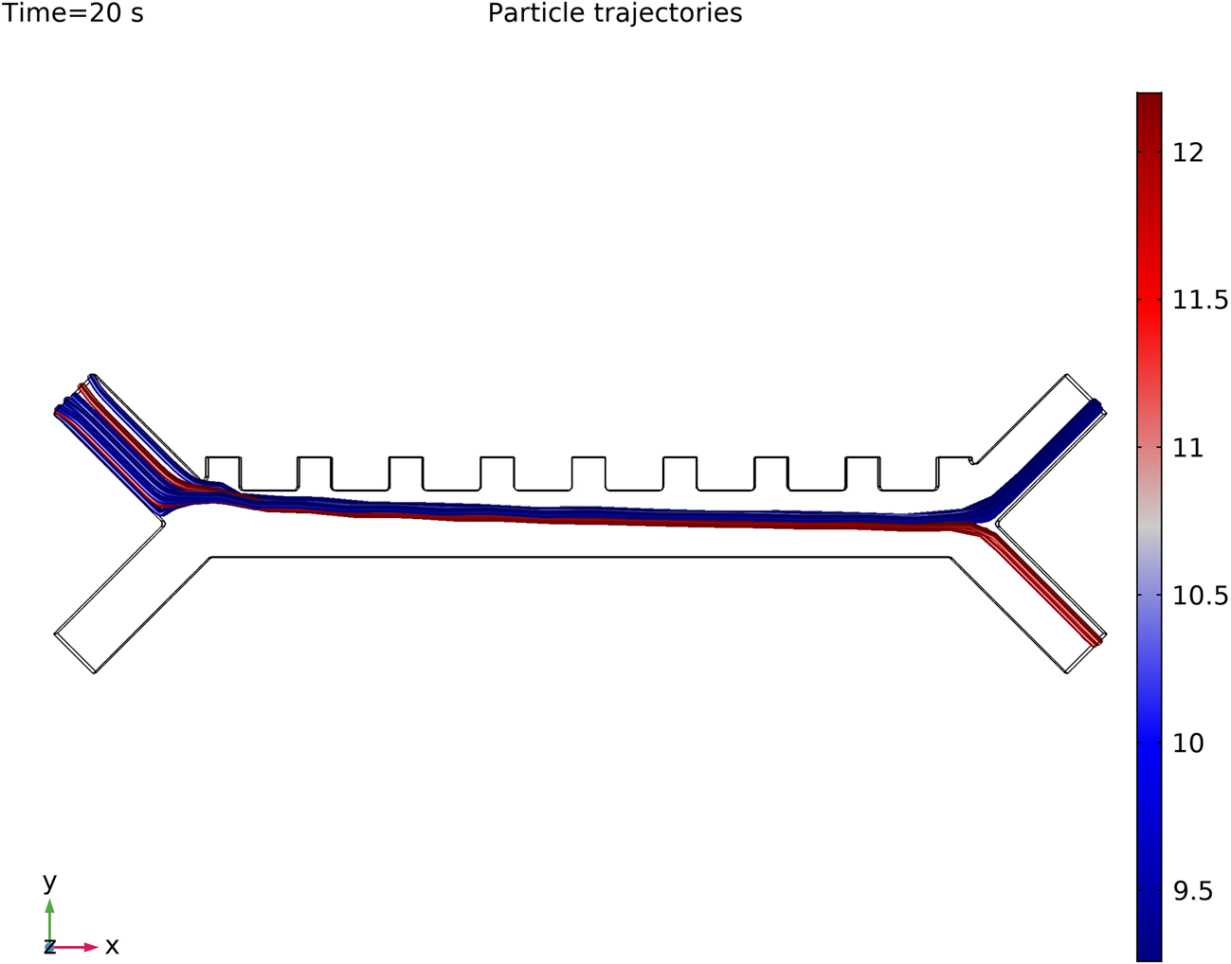
Particle Tracing result. Successful separation of CTC from leukocytes in the DEP stage. Cell suspension with random distribution of leukocytes and CTCs coming from the inertial stage enters into the inlet of the DEP stage. Color lines are cell trajectories, blue and its shades represent leukocytes while CTCs are represented by red color.

We investigated under pDEP that how significant the behavior difference between all the variants of leukocytes is by fixing applied frequency first at 350 kHz. The voltage could be varied in a wide range. Since cells are vulnerable to a high electric field, low working voltages are more desirable. Therefore, we examine the cell separation process using frequencies ranging between 350–400 kHz and voltages between 2–5 V. It is observed that to separate leukocytes from CTCs, for a given combination of voltage and frequency, a definite number of electrodes are required. The use of nine electrodes ensured sufficient separation at a frequency of 370 kHz Fig. 11 illustrates the final trajectories of particles which shows 99.5% separation of CTCs from leukocytes or WBCs Video 1. A very similar type of findings about breast cancer have been reported by moon and colleagues; in the similar direction colorectal cancer and lukemia have also been reported earlier that support our present findings by^39,82–84^.

## Conclusion

This study demonstrates the design and simulation of a highly specific, continuous, label-free two-stage microfluidic sorting device based on inertial focusing and DEP to isolate CTCs (hGBM) from whole blood. Present work aims to resolve the limitations of the DEP method by adding a pre-enrichment size-based separation stage. In the pre-enrichment stage small cells (typically RBCs and Plts) make most of the blood volume i.e., 45% are separated. These cells are responsible for blood conductivity and non-Newtonian nature. Separating them in the initial phase increases the isolation purity and efficiency of CTCs. The remaining leukocytes and CTCs having comparable sizes (9.26–12.2 µm) were transported to the DEP stage where separation is carried out based on electrical properties of the aforementioned cells. Resulting CTCs are viable and can be further utilized for diagnosis and in devising patient-specific therapy,thus, making the proposed methodology more useful. Results show a 99.5% purity level with a high throughput of 12.2 ml/hr. To enhance the separation efficiency, several computational simulations were done to verify our proposed design and integration. This integrated device simulation demonstrated the efficient sorting of the cells using our two-stage inertial-DEP device. In the first stage, small RBCs and Plts were separated from the blood samples using the inertial focusing technique. Large cells like CTCs and leukocytes were separated from each other using DEP force. Most of the researchers used spiked or binary samples for testing which incurs an inherent limitation for clinical practices. We have used a whole blood sample along with PBS with an admissible dilution ratio of 1:10. We have used hGBM as CTC in our investigation for which no DEP separation has been reported to the best of our knowledge. However, the proposed device can be used to isolate other types of tumor cells (provided size comparable to leukocytes) by changing design parameters of the DEP stage i.e., applied frequency and voltage.

The proposed device design had a concept to overcome the problem of cell loss, cell fragmentation, cytotoxicity and cell deformation which occurred during sample preparation for most types of cell separation methods. The study used inertial flow separation method instead of centrifugation. Usually, centrifugation is done as a prerequisite for preparing clinical or lab testing samples which causes above mentioned problems. As leukocytes and most of the CTCs have similar size therefore inertial method is not very popular for CTC sorting due to WBC or leukocytes contamination. The present study used label free, DEP method which sort different cell types according to their motion in response to the DEP field. This is due to the cell crossover frequency differences by virtue of their unique dielectric properties. The study employs two stage method thereby reducing the cell population, also low voltage and frequency reduce the possibility of joule heating and cell lysis. Although this method has high isolation efficiency it suffers from low processing volume, possibility of cell clogging due to overloading and joule heating because of applied voltages. The overall limited processing volume is due to the serial integration. Our device design offers an overall isolation efficiency or cell recovery of up to 99.5%. Overall our present simulation study has the potential for highly efficient device however, the genetic based variations in the sample, variation due to race and presence of other diseases as well as medicinal therapies that may affect the sample’s consistency, viscosity, cellular toxicity etc.

## Supplementary Information

Supplementary Legends.

Supplementary Video 1.

## Data Availability

The data that support the findings of this study are available from the corresponding author upon reasonable request.
