# Supplementary material for: Design of a novel integrated microfluidic chip for continuous separation of circulating tumor cells from peripheral blood cells
Source: Sci Rep. 2022 Oct 11;12:17016. doi: 10.1038/s41598-022-20886-1 (PMC9554048; doi:10.1038/s41598-022-20886-1)
Supplement: Supplementary file 1 — Supplementary Legends. [file 41598_2022_20886_MOESM1_ESM.docx]

**Video 1:**

Separation of CTC from the blood showing the particle trajectories as the blood sample moves along the microfluidic channel ;Video shows the DEP stage sorting the CTCs and remaining blood cells, coming from (upper inlet), on the basis of their dielectric properties. From the lower inlet buffer is injected, as cells enter the main channel, the buffer keeps the cells from getting to the bottom to avoid cell surface interaction and subsequent adhesion, due to the applied voltage and frequency, blood cells (particles in blue) experience positive dielectrophoretic force and they tend to be close to the electrodes, while CTCs (Red) experience negative DEP force and move away from the electrodes, eventually separating from blood cells then the isolated CTCs are collected in the lower channel, while blood cells including granulocyte and monocyte are separated in the upper waste channel.
